# The QKI-6 RNA Binding Protein Localizes with the MBP mRNAs in Stress Granules of Glial Cells

**DOI:** 10.1371/journal.pone.0012824

**Published:** 2010-09-17

**Authors:** Yunling Wang, Geneviève Lacroix, Jeffery Haines, Evgueni Doukhanine, Guillermina Almazan, Stéphane Richard

**Affiliations:** 1 Terry Fox Molecular Oncology Group, Lady Davis Institute for Medical Research and Departments of Oncology and Medicine, Bloomfield Center for Research on Aging, McGill University, Montréal, Québec, Canada; 2 Department of Pharmacology and Therapeutics, McGill University, Montréal, Québec, Canada; Emory University, United States of America

## Abstract

**Background:**

The *quaking viable* (*qk^v^*) mouse has several developmental defects that result in rapid tremors in the hind limbs. The *qkI* gene expresses three major alternatively spliced mRNAs (5, 6 and 7 kb) that encode the QKI-5, QKI-6 and QKI-7 RNA binding proteins that differ in their C-terminal 30 amino acids. The QKI isoforms are known to regulate RNA metabolism within oligodendrocytes, however, little is known about their roles during cellular stress.

**Methodology/Principal Findings:**

In this study, we report an interaction between the QKI-6 isoform and a component of the RNA induced silencing complex (RISC), argonaute 2 (Ago2). We show in glial cells that QKI-6 co-localizes with Ago2 and the myelin basic protein mRNA in cytoplasmic stress granules.

**Conclusions:**

Our findings define the QKI isoforms as Ago2-interacting proteins. We also identify the QKI-6 isoform as a new component of stress granules in glial cells.

## Introduction

The *quaking viable (qk^v^)* mice represent an animal model with dysmyelination defects in the central and peripheral nervous system (CNS, PNS) [1]. *Qk^v^* mice develop normally until about 10 days after birth at which time they display a rapid tremor that is especially pronounced in the hind limbs with adult mice also displaying tonic-clonic seizures [1]. The CNS defect is summarized as a lack in maturation of the myelin sheath or the arrest of myelinogenesis [2]. Oligodendrocyte differentiation is interrupted in *qk^v^* mice [1].

The genetic defect of the *qk^v^* mice has been linked to the *qkI* gene known to express 3 major alternatively spliced mRNAs of 5, 6 and 7 kb [3]. These mRNAs encode QKI-5, QKI-6 and QKI-7 respectively, which differ in their C-terminal 30 amino acids. The nuclear isoform, QKI-5, harbors a C-terminal nuclear localization signal (NLS) that is absent in the cytoplasmic isoforms QKI-6 and QKI-7 [4]. The expression of QKI-5 is highest during embryogenesis and the expression of cytoplasmic QKI-6 and QKI-7 isoforms is elevated during myelination. Part of the *qk* enhancer/promoter is deleted in the *qk^v^* mice [5] and as a result, QKI-6 and QKI-7 isoforms are not properly expressed in oligodendrocytes, as analyzed by immunocytochemistry [6]. It is thought that the inadequate expression of QKI-6 and QKI-7 causes the lack of oligodendrocyte maturation and the dysmyelination defects observed in *qk^v^* mice [6]. Consistent with this hypothesis, the forced expression of QKI-6 and QKI-7 induces oligodendrocyte and Schwann cell differentiation and maturation [7], [8]. Moreover, a transgenic allele that expresses Flag-QKI-6 specifically in the oligodendroglia lineage, driven by the proteolipid protein (PLP) promoter rescued the severe tremor and hypomyelination phenotype, when introduced into the *qk^v^* mice background [9].

The QKI proteins belong to the heteronuclear ribonucleoprotein particle K (hnRNP K) homology (KH) domain family of RNA binding proteins that bind specific RNA sequences with high affinity [10], [11], [12]. The QKI KH domain is embedded in a larger conserved domain often called the maxi-KH, the GSG (GRP33, Sam68, GLD-1) or the STAR (Signal Transduction and Activator of RNA) domain [12], [13]. Several post-transcriptional functions have been attributed to the QKI proteins. The QKI isoforms regulate the localization and export of myelin basic protein (MBP) mRNAs [14]. Moreover, the QKI isoforms regulate the stability of MBP [15] and p27^KIP1^ mRNAs [7]. The QKI isoforms have been proposed to function in alternative splicing in the myelin associated glycoprotein mRNA [16].

In the present manuscript, we define an interaction between the QKI isoforms and argonaute 2 (Ago2). Ago2 is a core component of the RISC complex which regulates gene expression including mRNA degradation and translation repression [17]. We also show that QKI-6 co-localizes with Ago2, PABP1, TIA1 and MBP mRNA in stress granules in glial cells. These findings identify the QKI-6 isoform as a new component of stress granules in oligodendrocytes.

## Results

### The QKI isoforms associate with Ago2

**Figure 1:**
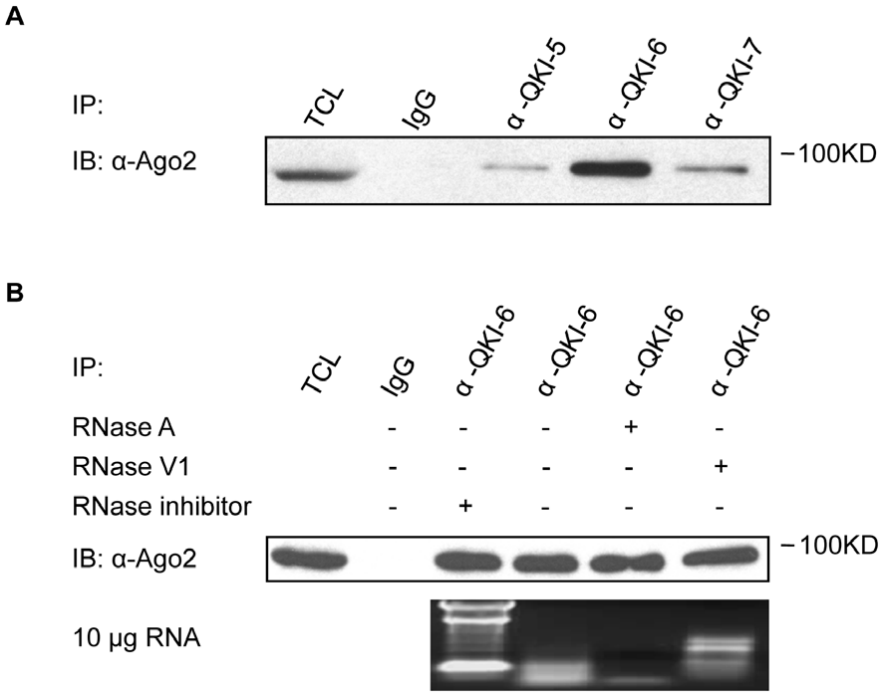
The endogenous QKI isoforms associate with Ago2. (A) U343 cell lysates were subjected to immunoprecipitations (IP) with control immunoglobulin G (IgG), anti-QKI-5, -QKI-6 and -QKI-7 antibodies. The bound proteins were separated by SDS-PAGE and immunoblotted (IB) with anti-Ago2 antibodies. (B) U343 cell lysates were treated with 1 mg/ml RNase A, 2 U/100 µl RNase V1 or RNase inhibitor as indicated at 37°C for 1 hr and subjected to immunoprecipitation with the anti-QKI-6 antibody. The proteins were separated by SDS-PAGE and immunoblotted with anti-Ago-2 antibodies as indicated (upper panel). The activity of the RNases and the RNase inhibitor was verified by agarose gel electrophoresis with 10 µg of total RNA (lower panel).

The QKI RNA binding proteins have recently been shown by photoactivatable-ribonucleoside-enhanced crosslinking and immunoprecipitation (PAR-CLIP) to associate with clusters of non-coding intronic RNAs [18]. Therefore, we reasoned that the QKI isoforms may associate with components of the RNA-induced silencing complex (RISC). To examine whether the QKI isoforms associate with Ago2, a member of the RISC complex, we performed co-immunoprecipitations in the U343, a human glioblastoma cell line known to express the three major QKI isoforms (QKI-5, -6, and -7). Cellular extracts were prepared and the QKI isoforms were immunopurified using anti-QKI-5, -6 and -7 antibodies and the bound proteins were separated by SDS-PAGE followed by immunoblotting with anti-Ago2 antibodies. Ago-2 was detected by immunoblotting in anti-QKI-5, -6 and -7 antibody immunoprecipitations (Figure 1A). Next we focused on QKI-6, as it had the highest relative affinity for Ago2. To determine whether the QKI-6 and Ago2 interaction was RNA-dependent, U343 cell lysates were pre-incubated with RNase A or V1, the enzymes that digest ssRNA and dsRNA, respectively, before performing the immunoprecipitations. The QKI/Ago2 interaction was insensitive to high doses of RNase A or V1 (Figure 1B), suggesting that the interaction is likely mediated by a protein-protein interaction and not by a bridging RNA.

**Figure 2:**
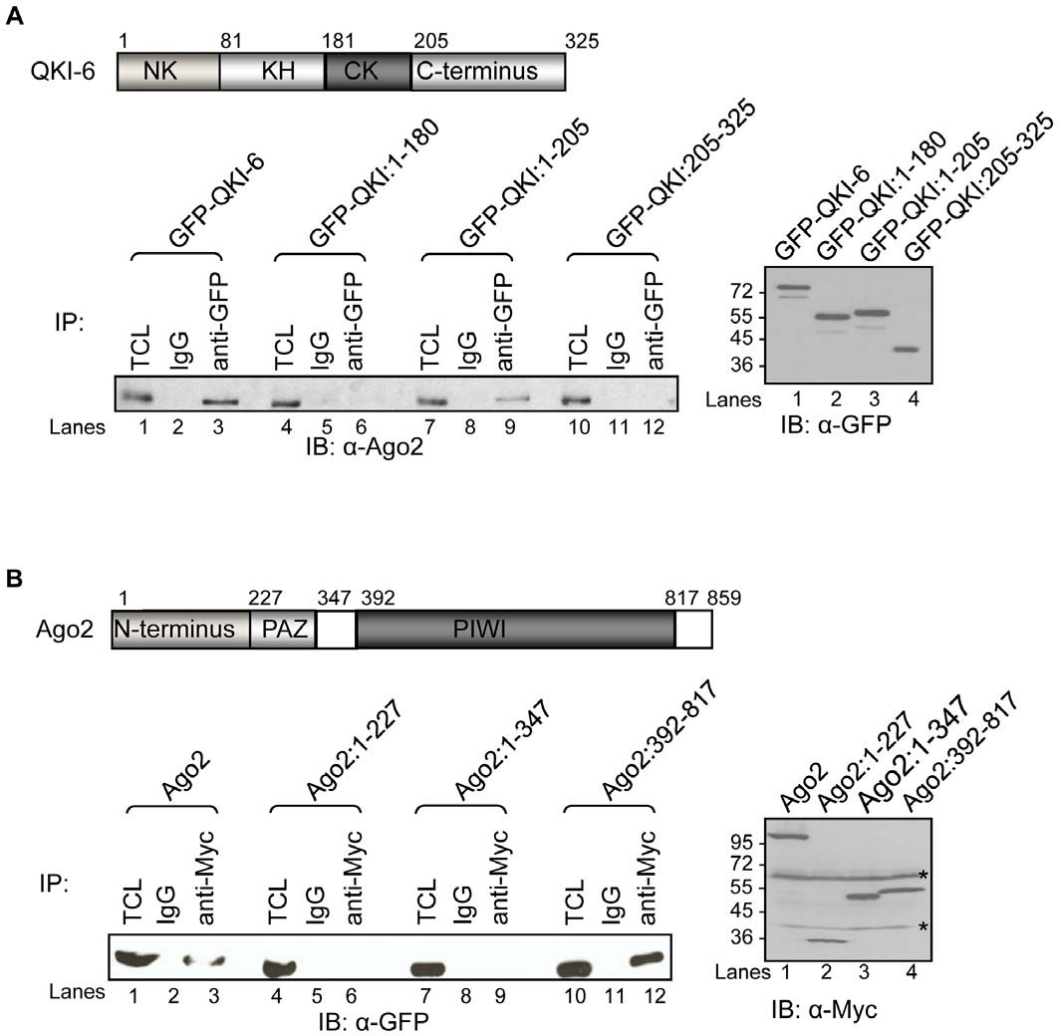
Mapping the domains required for the QKI-6/Ago2 interaction. (A) A schematic illustration of the QKI-6 protein showing its regions and its amino acid numbering. Expression vectors encoding GFP-QKI-6 and truncation mutants thereof were transfected in HEK293 cells. The transfected cells were lysed and the cell lysates were subjected to immunoprecipitation with the anti-GFP antibody and the bound proteins separated by SDS-PAGE. The presence of Ago2 was monitored by using anti-Ago2 antibodies as indicated (left panel). Extracts before immunoprecipitation were separated by SDS-PAGE and immunoblotted with anti-GFP antibodies to confirm equivalent expression. The molecular mass markers are shown on the left in kDa. (B) A schematic illustration of Ago2 is shown with its conserved domains and the numbering of its residues. The GFP-QKI-6 expression plasmid was cotransfected with myc-tagged full-length Ago2 or truncation mutants in HEK293 cells, as indicated. Twenty four hours after transfection, the cells were lysed and cell lysates were subjected to immunoprecipitation with anti-Myc antibodies followed by immunoblotting with anti-GFP antibodies. The migration of GFP-QKI-6 is shown (left panel), while the expression of the myc-Ago2 proteins is shown in the right panel. The asterisks denote non-specific proteins recognized by the anti-myc antibodies. The molecular mass markers are shown on the left in kDa.

To identify the regions required for the interaction, QKI-6 and Ago2 truncated proteins were expressed in HEK293 cells followed by co-immunoprecipitation assays. The QKI isoforms harbor a KH domain flanked by the N- (NK) and C- (CK) terminal regions (Figure 2A), while Ago2 contains an N-terminal region (N-terminus), as well as PAZ and PIWI domains (Figure 2B). Myc-Ago2 co-immunoprecipitated with GFP-QKI:1-205, but not with GFP-QKI:205–325 or GFP-QKI:1–180 (Figure 2A), revealing that the QKI-6 CK region was required for the interaction with Ago2. Moreover, GFP-QKI-6 co-immunoprecipitated with myc-Ago2:392–817, but not myc-Ago2:1–227 or myc-Ago2:1–347 (Figure 2B), indicating that the Ago2 PIWI domain was necessary and sufficient for interaction with QKI-6.

**Figure 3:**
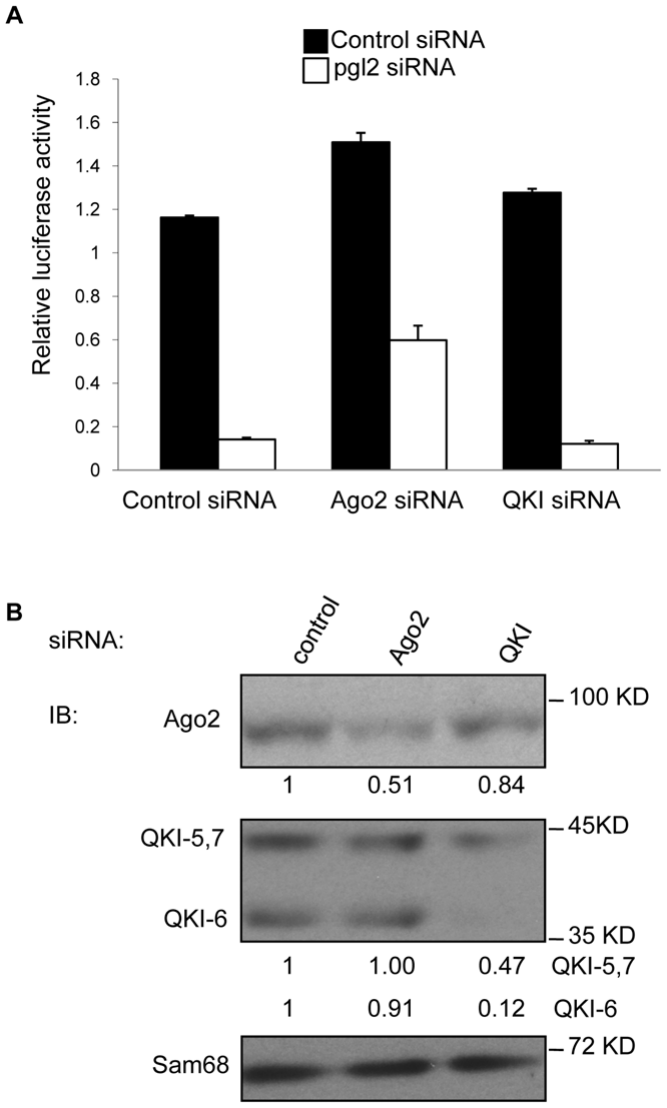
RNA interference is not affected by the absence of the QKI-6 isoform in U343 cells. (A) Control siRNA, siQKI-6 or siAgo2 were transfected in U343 cells and 24 hr later, the pMIR luciferase plasmid was co-transfected with the renilla luciferase vector and an siRNA that targets the pgl2 against firefly luciferase or a control siRNA. Thirty hours later, the cells were lysed and subjected to luciferase assays or immunoblotting analysis panel B. The firefly luciferase activity was normalized with renilla luciferase. (B) Cellular extracts from transfected U343 cells from panel (A) were immunoblotted with anti-Ago2 and anti-QKI antibodies. Immunoblotting against anti-Sam68 antibodies was used as a loading control. Absolute quantified levels of protein bands are presented underneath each blot and were performed using Scion-Image.

To investigate whether the QKI isoforms are key accessory proteins of Ago2 in the RISC complex, we reasoned that reduced expression of the QKI isoforms would impair the RNA interference of a luciferase reporter gene. U343 cells were transfected with siRNAs targeting the QKI isoforms, a negative control siRNA targeting GFP or a positive control siRNA targeting Ago2. Twenty-four hours later, the cells were transfected with a luciferase reporter plasmid along with a control siRNA or a luciferase siRNA and luciferase activity was assessed 30 h later. Ago2 knockdown significantly impaired the ability of the luciferase siRNA to function, whereas the knockdown of the QKI isoforms had no effect (Figure 3). These results suggest that the QKI isoforms are not components of the RISC complex and that the QKI/Ago2 interaction fulfills other functions.

### QKI-6 and QKI-7 co-localize with Ago2 in cytoplasmic granules upon oxidative stress treatment

**Figure 4:**
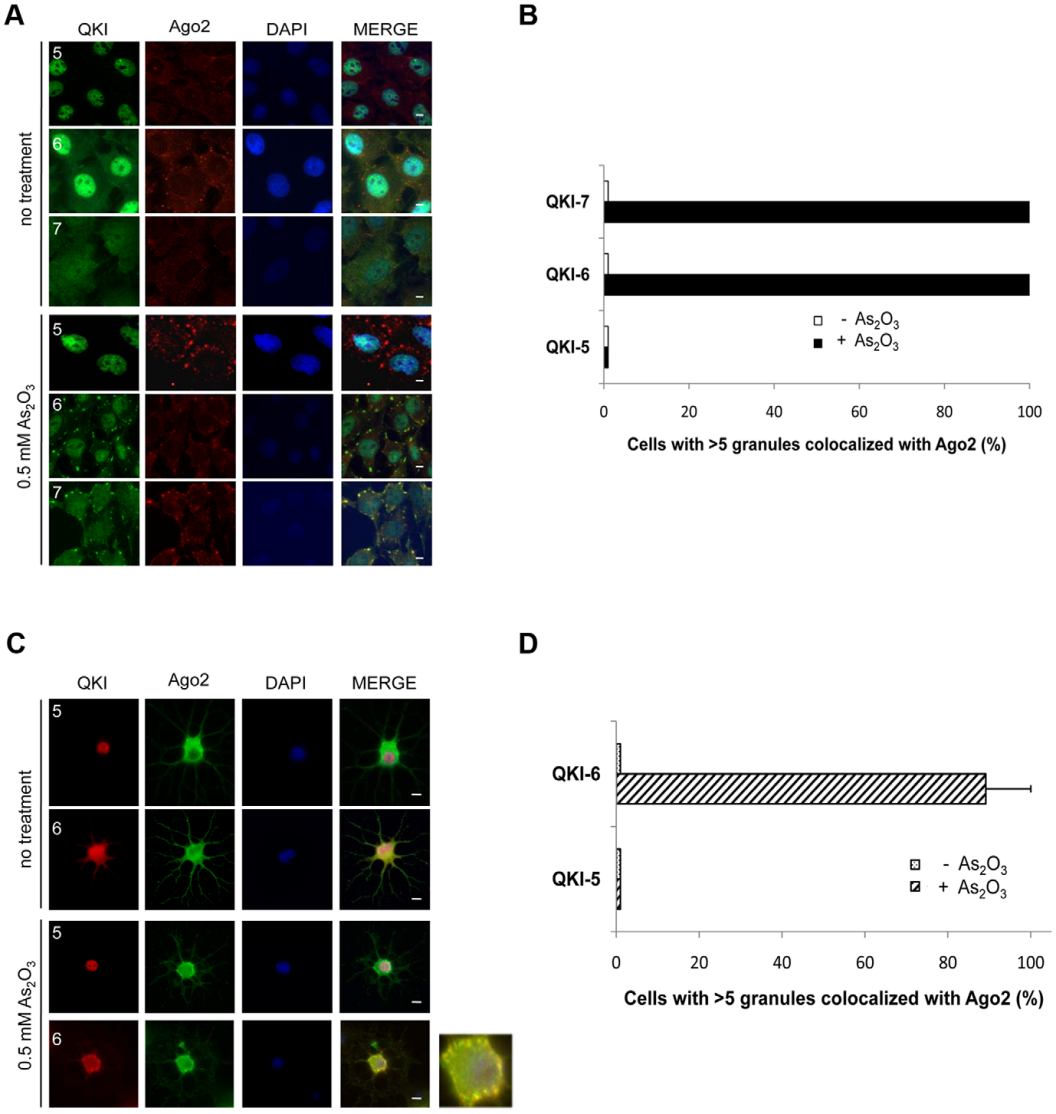
QKI-6 and QKI-7 isoforms co-localize with Ago2 in cytoplasmic granules in glial cells. (A) U343 cells were untreated or treated with 0.5 mM arsenic oxide (As_2_O_3_) for 45 min. The cells were fixed, permeabilized and immunostained with rabbit anti-QKI-5, -6 and -7 antibodies and mouse anti-Ago2 antibodies followed by secondary goat anti-rabbit Alexa 488 (green) and goat anti-mouse Alexa 523 (red) antibodies. The nuclei were counter-stained with DAPI. The scale bar represents 5 µm. (B) The quantification of the co-localization between the QKI isoforms and Ago2 expressed as a percentage is shown. (C) Primary rat oligodendrocytes were untreated or treated with 0.5 mM As_2_O_3_ for 45 min and analyzed as in panel (A). (D) The quantification was performed as in panel (B).

**Figure 5:**
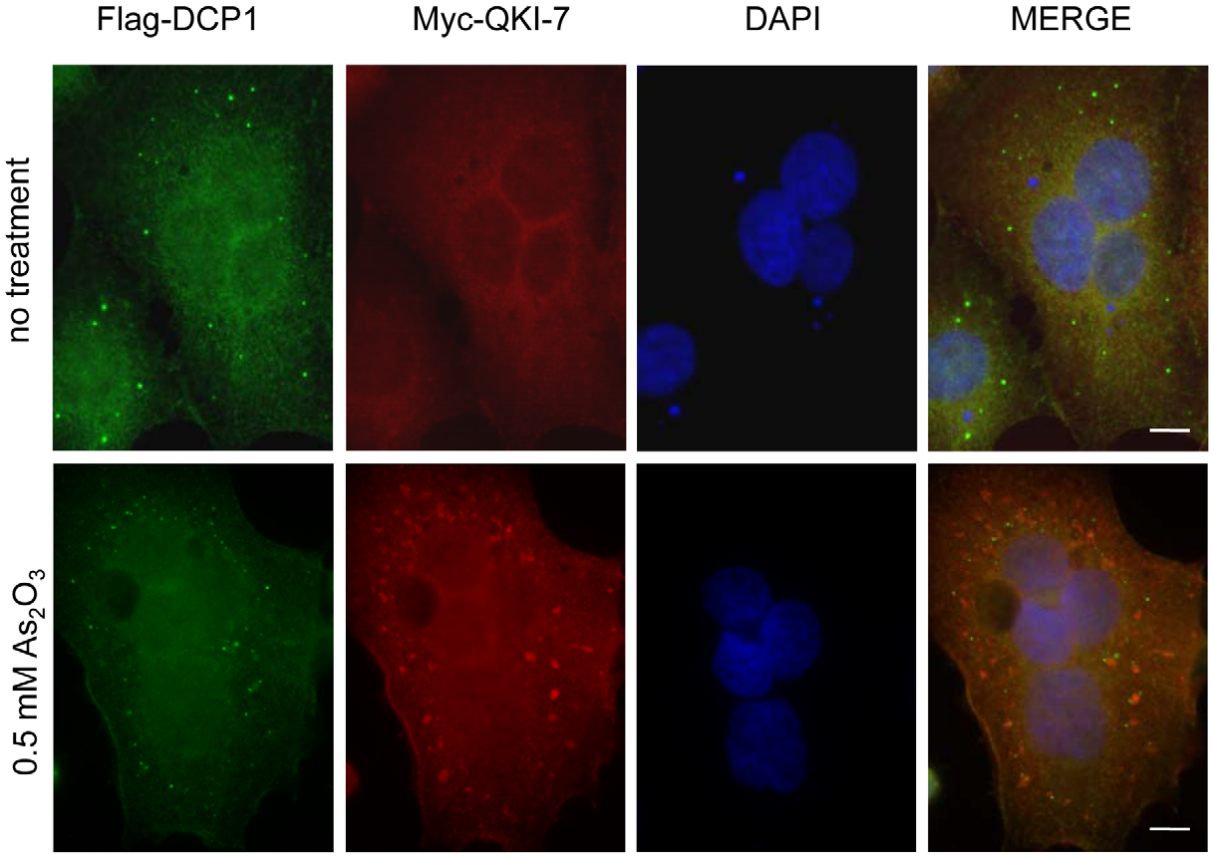
DCP-1 P bodies are devoid of QKI-7. Myc-QKI-7 and Flag-DCP1 were co-transfected in HEK293 cells. Thirty hours later, the cells were left untreated or treated with 0.5 mM As_2_O_3_ for 30 min. The cells were fixed, permeabilized and immunostained with rabbit anti-Myc antibody and mouse anti-Flag antibody followed by goat anti-rabbit Alexa523 (red) and goat anti-mouse Alexa 488 (green). The nuclei were counter-stained with DAPI. The scale bar represents 10 µm.

We examined whether QKI-5, QKI-6 and QKI-7 co-localize with Ago2 in U343 cells. In the absence of any treatment, Ago2 did not co-localize significantly with any of the QKI isoforms (Figure 4A). Endogenous QKI-5 was exclusively nuclear as expected (Figure 4A), while QKI-6 and QKI-7 were localized diffusely throughout the cell with QKI-6 also concentrating in the nucleus, as detected in U343 cells using anti-QKI-6 antibodies (Figure 4A). Endogenous Ago2 was localized diffusely in the cell and accumulated in discrete cytoplasmic foci termed RNA processing bodies or P bodies (Figure 4A). However, P bodies were devoid of the QKI isoforms (Figure 4A) and the QKI-7 isoform did not co-localize with DCP1 in P bodies (Figure 5).

Ago2 is known to re-localize to cytoplasmic granules under stress [19]. Therefore we performed the co-localization studies in cells treated with 0.5 mM arsenic oxide to induce oxidative stress. The treatment of U343 cells with arsenic oxide induced Ago2/QKI-6 and Ago2/QKI-7 co-localization within cytoplasmic granules in essentially 100% of the cells (Figure 4A, 4B). The nuclear isoform (QKI-5) did not co-localize with Ago2 demonstrating isoform specificity (Figure 4A, 4B). We next examined whether Ago2 and the QKI isoforms co-localized in normal differentiated primary rat oligodendrocytes. Ago2 co-localized in >90% of the cells with QKI-6 in cytoplasmic granules in oligodendrocytes treated with arsenic oxide (Figure 4C, 4D). Ago2 did not co-localize with the nuclear QKI-5 isoform, as expected (Figure 4C, 4D). These findings suggest that Ago2 localizes with QKI-6 and QKI-7 in cytoplasmic granules of glial cells.

### QKI-6 and QKI-7 co-localize with PABP1 and TIA1 within stress granules

**Figure 6:**
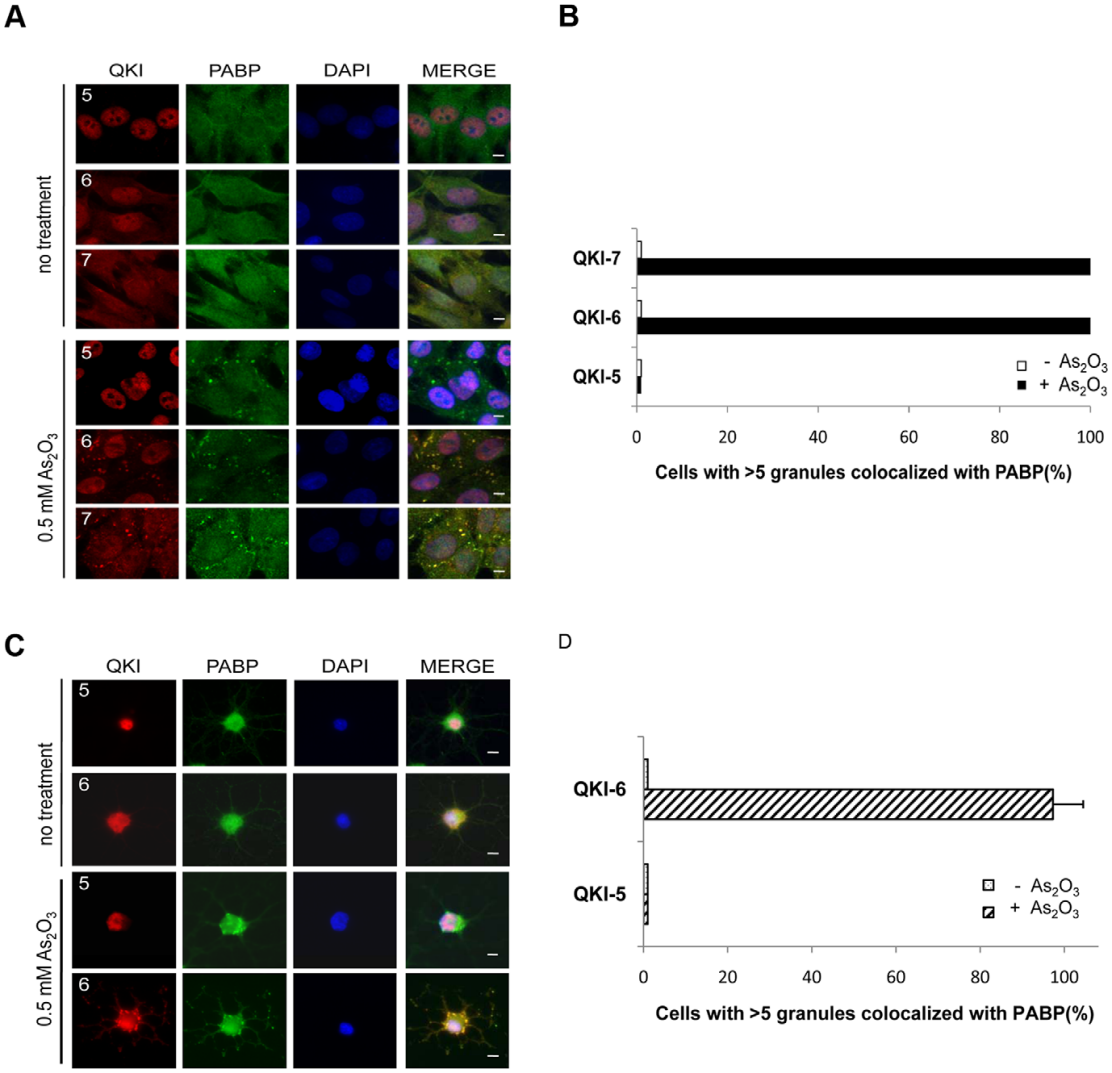
QKI-6 and QKI-7 co-localize with PABP1 in stress granules in glial cells. (A) U343 cells were untreated or treated with 0.5 mM As_2_O_3_ for 45 min. The cells were fixed, permeabilized and immunostained with rabbit anti-QKI-5, -6 and -7 antibodies and a mouse anti-PABP1 followed by secondary goat anti-rabbit Alexa523 (red) and goat anti-mouse Alexa 488 (green) antibodies. The nuclei were stained with DAPI. The scale bar represents 5 µm. (B) The quantification of the co-localization between the QKI isoforms and PABP1 expressed as a percentage is shown. (C) Primary rat oligodendrocytes were untreated or treated with 0.5 mM As_2_O_3_ for 45 min and analyzed as in panel (A). (D) The co-localization was performed as in panel (B).

**Figure 7:**
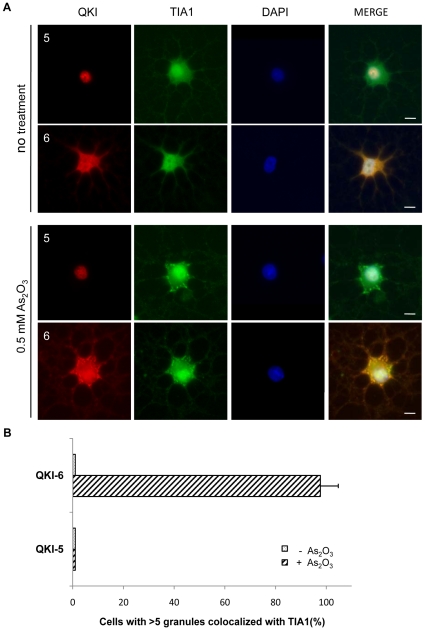
QKI-6, but not QKI-5 co-localizes with TIA1 in stress granules in primary rat oligodendrocytes. (A) Primary rat oligodendrocytes were untreated or treated with 0.5 mM As_2_O_3_ for 45 min. The cells were fixed, permeabilized and immunostained with rabbit anti-QKI-5, or -6 antibodies and goat anti-TIA1 antibody followed by secondary donkey anti-rabbit Alexa523 (red) and donkey anti-goat Alexa 488 (green) antibodies. The nuclei were counter-stained with DAPI. The scale bar represents 5 µm. (B) The quantification of the co-localization between the QKI isoforms and TIA1 expressed as a percentage is shown.

To confirm that QKI-6 and QKI-7 are components of stress granules, we tested under conditions of stress, whether the QKI-6 and QKI-7 isoforms co-localize with the poly A-binding protein (PABP1), a known marker of stress granules [20]. Indeed, all of the U343 cells and ∼95% of the primary rat oligodendrocytes exhibited PABP1/QKI-6 and PABP1/QKI-7 co-localization in stress granules upon arsenic oxide treatment (Figure 6). No significant co-localization between PABP1 and the QKI isoforms was observed in the absence of stress (Figure 6). We subsequently examined whether another marker of stress granules, TIA1, also co-localized with QKI-6. In ∼90% of the primary rat oligodendrocytes treated with arsenic oxide, TIA1 co-localized with QKI-6, but not QKI-5 (Figure 7), demonstrating that the QKI-6 isoform is a component of stress granules in glial cells.

### The MBP mRNA, a known QKI target, localizes within stress granules of primary rat oligodendrocytes

**Figure 8:**
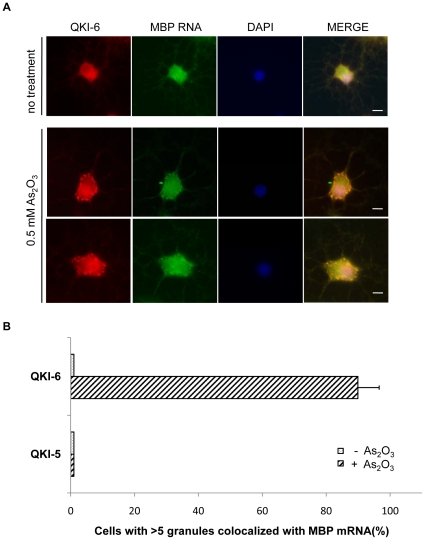
QKI-6 co-localizes with MBP mRNA in stress granules in primary rat oligodendrocytes. (A) Primary rat oligodendrocytes were left untreated or treated with 0.5 mM As_2_O_3_ for 45 min. The cells were fixed and hybridized with a digoxigenin-11-UTP labeled MBP antisense RNA probe (green) and immunostained with anti-QKI-6 antibodies (red). The scale bar represents 5 µm. (B) The quantification of the co-localization between the QKI isoforms and MBP mRNA expressed as a percentage is shown.

Cellular stress induces rapid shuttling of proteins and RNAs within stress granules [20]. Their presence within this structure is reversible, as stress granules are not sites of long-term mRNA ribonucleoprotein (mRNP) storage [20]. To examine if cellular stress regulates the ability of certain QKI mRNA targets to localize within stress granules, *in situ* hybridization was performed to test whether MBP mRNA co-localize with the QKI-6 isoform during stress. The MBP mRNA is a known QKI target with specific binding sites within its 3′-untranslated region [14], [15], [21], [22]. In the oligodendrocytes, the expression of QKI-6 and QKI-7 facilitates the mRNA export of MBP mRNAs [14], suggesting that the QKI RNA binding proteins serve to export certain mRNA targets. Using primary rat oligodendrocytes, we observed that ∼85% of cells harbor >5 foci that colocalize the MBP mRNA and QKI-6 in primary rat oligodendrocytes treated with arsenic oxide (Figure 8). Our results show that QKI-6 and the MBP mRNAs localize in stress granules in oligodendrocytes.

## Discussion

In the present study, we show that the cytoplasmic QKI isoforms, QKI-6 and QKI-7, but not nuclear isoform (QKI-5), localize with Ago2, PABP1, and TIA1 within stress granules in the U343 glioblastoma cell line and in primary rat oligodendrocytes. Interestingly, the MBP mRNA, a known QKI RNA target [14], [15], also localized in stress granules of primary rat oligodendrocytes. These findings identify the QKI-6 isoform as a new component of stress granules in oligodendrocytes.

In response to environmental stress, mRNAs are transiently transported to stress granules [23], [24]. Interestingly, a component of the RISC complex, Ago2, also accumulates in stress granules, but its role is unknown in these structures [19]. It is known that translational repression occurs in both stress granules and P bodies, while RNA degradation occurs mainly in P bodies [20]. Our observations that QKI-6 and QKI-7 co-localize with Ago2 in stress granules, but not in P bodies, suggesting that QKI-6 and QKI-7 are components of stress granules. In primary rat oligodendrocytes there is precedence for this with the Staufen RNA binding protein [25]. We now extend these findings and show that the QKI-6 isoform is a new component of stress granules in oligodendrocytes.

The QKI RNA binding proteins are involved in various aspects of RNA processing including mRNA stability, mRNA export, and pre-mRNA splicing [1], [26]. Ago2 is mainly known for its role as a component of the RISC complex and its involvement in miRNA-dependent translational repression [17]. The knockdown of the QKI isoforms in U343 cells did not affect the RNA interference function of Ago2 (Figure 3), suggesting that the QKI proteins are not key components of the RISC complex. Previous reports showed that fragile X mental retardation 1 (FMR1), an RNA binding protein, is required for Ago2-mediated translational regulation [27]. It was shown that the fragile-X-mental-retardation-related protein 1 (FXR1) and Ago2 associate with an AU-rich elements in mRNAs exclusively during activation of translation under serum starvation [28]. Whether the QKI-6 fulfills a similar function to FXR1 in conjuction with Ago2 remains to be investigated.

RNA localization elements are recognized by specific RNA-binding proteins regulating many aspects of RNA metabolism [29], [30]. The mRNAs of many myelin components, such as MBP, are translationally repressed and transported to the distal tips of the oligodendrocyte processes to be locally translated and directly incorporated into growing myelin sheath [31], [32]. QKIs regulate the nuclear export of MBP mRNAs by associating with a specific high-affinity QKI response element within the 3′-untranslated region [14], [22], [33]. These findings identify the QKI-6 isoform as a new component of stress granules in oligodendrocytes.

## Materials and Methods

### Cells and transfections

The HEK293 and U343 cell lines were purchased from American Type Culture Collection (Manassas, VA) and primary oligodendrocyte precursor cells were prepared as described previously [34]. To induce oxidative stress, cells were treated with 0.5 mM arsenic trioxide (catalog number A1010, Sigma, St. Louis, MO) for 45 min as described previously [35]. Plasmids were transfected with Lipofectamine™ 2000 (Invitrogen) and siRNAs were transfected with Lipofectamine™ RNAiMAX (Invitrogen) according to the manufacturer's instructions.

### Antibodies and plasmids

Anti-Sam68 and anti-QKI-5, -6 and -7 antibodies were purchased from Millipore Inc. Antibodies against β-actin and Flag were purchased from Sigma Inc. The anti-Ago2 antibody was purchased from Novus Biologicals. The Myc-Ago2 pcDNA was kindly provided by Gregory J. Hannon (Cold Spring Harbor, NY) [36]. Myc-Ago2:1–227, myc-Ago2:1–334, and myc-Ago2:392–817 were generated by PCR with myc-Ago2 as DNA template. The sequences of the oligonucleotide pairs used were 5′-CACCATGGAGCAAAAGCTCATCTCAG-3′ and 5′-CTATGCCTTGTA TAAAACGCTGTTG-3′ for myc-Ago2:1-227; 5′- CACCATGGAGCAAAAGCTCAT CTC AG-3′ and 5′-CTACACAATGTTACAGACCTCCAG-3′ for myc-Ago2:1-334; and 5′-CACCATGGAGCAGAAACTCATCTCTGAAGAGGATCTTGT-3′ and 5′- CTACAGGTGGTACCTGGCCCGGAAG-3′ for myc-Ago2:392-817. The amplified PCR fragments were sub-cloned into the pcDNA™3.1 Directional TOPO® Expression vector (Invitrogen) following the manufacturer's procedure. The GFP-QKI:1–180, GFP-QKI:1–205 were described previously [37]. The GFP-QKI-6:205–325 was generated by using PCR with GFP-QKI-6 as DNA template [38]. The sequences of oligonucleotide pairs used were 5′- GCATGAATTCACCAGCCCTTGCGTTTT C-3′ and 5′-TACAAATGTGGTATGGCTGA-3′. The amplified DNA fragment was digested with *Eco*RI and *Bam*HI and sub-cloned into the vector pEGFP-C1 (Clontech).

### Immunoprecipitation and RNase treatment

U343 cells or transfected HEK 293 cells were harvested and lysed in Triton X-100 lysis buffer, as described previously [7]. The extracts were treated with RNaseA (Boehringer Mannheim) at 1 mg/ml for ssRNA digestion or RNaseV1 (Ambion) at 2 U/100 µl for dsRNA digestion at 37°C for 1 hr. The extracts without RNase treatment were considered as mock treatment. After the RNase treatment, the cell lysates were utilized to perform immunoprecipitations. The bound proteins were analyzed by immunoblotting with the indicated antibodies.

### Immunofluorescence

Cells were grown at 30 to 50% confluency and fixed with 4% paraformaldehyde in PBS for 30 min and permeabilized with 0.5% Triton X-100 in PBS for 10 min at room temperature. The permeabilized cells were incubated with primary antibodies in PBS for 2 hr at RT. The cells were washed 3 times with 0.1% Triton X-100 in PBS and incubated with the appropriate fluorescent secondary antibodies (Invitrogen) in PBS for 1 hr (Millipore Inc. 1∶200). Primary antibodies were diluted as follows: anti-QKIs rabbit polyclonal antibodies (1∶200), anti-Ago2 monoclonal antibody (Abnova, 1∶50), anti-PABP1 rabbit polyclonal antibody (Cell Signaling, 1∶25), anti-PABP1 mouse monoclonal antibody (Sigma, 1∶10), anti-TIA1 goat polyclonal antibody (Santa Cruz Biotechnology, 1∶50), anti-Flag monoclonal antibody (Sigma, 1∶100) and anti-Myc monoclonal antibody (Sigma, 1∶100).

### Fluorescent *in situ* hybridization (FISH)

A 351 base pair DNA fragment was amplified by PCR using an expression vector for the MBP 14 kDa isoform [14] with the following oligonucleotides 5′-ATGGCATCACAGAAGAGACC-3′ and 5′- TCTTCCTCCCCAGCTAAATC-3′. The DNA fragment was cloned into pBluescript KS+ vector. Antisense and sense UTP digoxigenin (Roche Applied Science) RNA probes were synthesized with T7 or T3 RNA polymerase *in vitro*. The *in situ* hybridization was performed with 5 ng/µl digoxigenin-riboprobe as described [14].

### Luciferase assay

U343 cells were transfected with control, Ago2 and QKI siRNAs using Lipofectamine™ RNAi Max (Invitrogen). Twenty-four hours after transfection, the cells were co-transfected with pMIR firefly luciferase (FL) reporter (Ambion) plasmid and pRL-TK renilla luciferase (RL) plasmid (Promega) as well as a control or pgl2 siRNA targeting the pMIR firefly luciferase. The FL/RL activities were measured 24 to 30 hr after the second transfection with the Dual-Luciferase® Reporter Assay system (Promega Inc.). In addition, cell extracts prepared and the proteins were separated on SDS polyacrylamide gels followed by immunoblotting. All the siRNAs were purchased from Dhamacon Inc with targeting sequences were: QKI; GGACUUACAGCCAAACAAC, Ago2; UGGACAUCCCCAAAAUUGA and pgl2; CGUACGCGGAAUACUUCGA.
